# A qualitative study exploring child marriage practices among Syrian conflict-affected populations in Lebanon

**DOI:** 10.1186/s13031-017-0131-z

**Published:** 2017-11-14

**Authors:** Rima Mourtada, Jennifer Schlecht, Jocelyn DeJong

**Affiliations:** 1 Faculty of Health Sciences, American University of Beirut, P.O. Box 11-0236/EPHD, Riad El Solh, Beirut, Lebanon; 2 Women’s Refugee Commission, 122 E 42nd Street, 11th Floor, New York, NY 10168 USA

**Keywords:** Conflict, Displacement, Syrian refugees, Early marriage, Child marriage, Lebanon

## Abstract

**Background:**

Recent reports have suggested that child marriage among Syrians may be increasing as a result of displacement and conflict. This study sought to gather qualitative data about the factors that promote child marriage practices among Syrian refugees in Al Marj area in the Bekaa valley, Lebanon, where the majority of Syrian refugees have settled in Lebanon. The second aim of this study was to generate recommendations on how to mitigate the drivers and consequences of child marriage practices based on the findings.

**Methods:**

Eight focus group discussions were conducted separately with married and unmarried young women and mothers and fathers of married and unmarried women. Furthermore, researchers conducted 11 key informant interviews with service providers and stakeholders to understand how conflict and displacement influenced marriage practices of Syrian refugees in Al Marj community.

**Results:**

Although child marriage was a common practice in pre-conflict Syria, new factors seem to contribute to a higher risk of child marriage among Syrian refugees in Lebanon. Respondents cited conflict- and displacement-related safety issues and feeling of insecurity, the worsening of economic conditions, and disrupted education for adolescent women as driving factors. Service providers, young women, and parents also reported changes in some marriage practices, including a shorter engagement period, lower bride price, change in cousin marriage practices, and a reduced age at marriage.

**Conclusions:**

Recommendations for interventions to mitigate the drivers of child marriage and its negative consequences should be built on a clear understanding of the local refugee context and the drivers of child marriage in refugee settings. Interventions should involve multiple stakeholders, they should be adjusted to target each specific context, age group and marital status. For these interventions to be effective, they should be addressed concurrently, and they should be delivered in a culturally sensitive and practical manner.

## Background

Child marriage, any marriage where one or both spouses are under the age of 18, presents a significant public health concern for adolescent girls and their communities. It undermines efforts to improve child health and survival, to reduce maternal mortality, and to achieve universal primary education. Ninety percent of early first births happen within the context of child marriage, and girls between 15 and 19 years of age are far more likely to experience complications during pregnancy and childbirth than those over twenty [1, 2]. Married adolescents face higher risks of intimate partner violence [3]. Additionally, child marriage increases exposure to sexually transmitted infections, including HIV, particularly when the age difference between the girl and her partner is significant [4–6]. In addition to health concerns, child marriage introduces other pernicious effects. Marriage often means the end of a girl’s education and vocational opportunities. Although the practice affects both sexes, girls are usually more affected than boys [1].

Fragility and conflict have been closely linked to child marriage practices. Child marriage and teen pregnancy appear to be particularly high in insecure environments [7–9]. Nine of the top 10 countries with the highest rates of child marriage are considered fragile states [8]. Similarly, many countries with particular vulnerability to natural disasters are among the highest in terms of child marriage prevalence [9].

Child marriage is receiving increased attention from the humanitarian actors. It is included as a component of the revised 2015 Interagency Standing Committee (IASC) guidelines on gender-based violence (GBV) [10]. Child marriage is also a concern for the Global Protection Cluster’s Child Protection Working Group. Agencies, including the Council on Foreign Relations, UNICEF, and World Vision, have identified factors that drive child marriage during conflict, including new protection concerns (such as threats of sexual violence), economic strain after displacement, and gaps in education and vocational opportunities [7, 11, 12].

Available literature on child marriage in conflict settings shows that conflict does not always lead to a rise in child marriage rates and its effect on child marriage also depends on economic and social factors. For instance, the age at marriage in both Lebanon and Algeria, which experienced a civil war and considerable deal of violence, is relatively high [13].

Child marriage practices among Syrian refugees in Lebanon and Jordan are reported to be high as a result of displacement and conflict [14–17]. Although not widespread in the contemporary Arab region, child marriage was a significant practice in Syria before the conflict. The percentage of women aged 20 to 24 years who were first married or in union before the age of 18 years was estimated at 13% over the period 2002–2011 by UNICEF [18], and at 17.3% in 2009 in the nationally representative Syria Family Health Survey [19]. Marriage is registered by religious courts in Syria and most religious groups in Syria allow girls under the age of 18 to marry with the permission of a legal guardian [14]- and the minimum age for marriage is 13 years of age [20].

Although it is challenging to ascertain the prevalence of child marriage in humanitarian or conflict settings through comprehensive surveys due to lack of marriage registration and difficulties in obtaining valid information about population size [13], it appears that a growing number of Syrian refugees are relying on child marriage practices to cope with the financial and protection challenges they are facing as refugees. A recent study found that, in Lebanon, 18% of Syrian refugee adolescent girls (15–18 years of age) were married [16]. Another survey found that the rate of child marriage among Syrian refugees in Lebanon was 23% [17]. Although neither survey is fully representative of the Syrian refugee population, the reported child marriage figures are higher than the pre-war figure inside Syria and may be indicative of change. However, such results should be interpreted with caution taking into account that refugees are often a self-selected group that does not represent the entire Syrian population. Additionally, such observed figures may not be even representative of the entire refugee population in those countries [13].

Given the scale of the current Syrian crisis, with more than 5 million Syrians displaced to neighboring countries [21], it is important to understand the extent to which child marriage may be affecting young adolescents and how local or humanitarian actors can ameliorate the risks and consequences of child marriage effectively.

The aim of this study was to gather qualitative data about the factors that promote child marriage practices among Syrian refugees in Al Marj area in Bekaa, where the majority of Syrian refugees are settled in Lebanon. The second aim was to generate recommendations on how to decrease child marriage practices based on the findings.

## Methods

### Study design

This research was qualitative in nature and took a phenomenological approach [22, 23], seeking to learn from the experiences that young women, parents and programmers, have had with marriage decisions both before and after the conflict. This study was implemented as a follow up to research examining the needs and risks of Very Young Adolescents, which was implemented in 2014, in three humanitarian contexts [24].

### Study setting

Lebanon hosts the largest proportion of Syrian refugees (1,011,366) in relation to the country’s population; 18.2% of those are girls aged 5–17 years of age [21]. The majority of Syrian refugees in Lebanon currently live in the Bekaa area (361,104), east of Beirut [21]. At the time of this research (November/ 2014), there were about 12,000 displaced Syrians in the study setting of Al Marj, Bekaa.

Refugees in Al Marj live either in tented settlements or in rented rooms or flats. Tented settlements, resembling very small refugee camps, are located on agricultural land, not far from town centers. Refugees pay between $75 and $100 per month to reside in the tented settlements, and have no formal tenancy agreements. Tents are small and are often shared by numerous families. This congestion encourages the spread of infectious diseases and may increase protection risks. The tents have limited access to clean water, and no appropriate sanitation facilities [25, 26].

Refugees who live outside the settlements are more likely to come from wealthier, urban areas, but pay higher rent: roughly $205 per month according to a national vulnerability assessment in 2015 [27].

The relationship between Syrian refugees and Lebanese nationals is complex and at times strained [28]. Local resources and services are unable to absorb the impacts of this massive influx [29]. Additionally, new power dynamics expose Syrians to possible exploitation, including sexual exploitation by property owners [28]. Syrians’ access to the labor market in Lebanon has been restricted since 2014, and 70 % of displaced Syrian refugees in Lebanon now live below the extreme poverty line [29]. In 2014, more than 50% of Syrian refugee children in Lebanon aged 5 to 17 years old were not enrolled in any form of education, with adolescents facing the largest obstacles [30].

Syrian refugees face a number of challenges when registering their marriages and births in Lebanon, which limits their access to services such as shelter and education [31, 32]. For instance, the main steps required to register a birth in Lebanon are: obtaining a birth notification from the birth attendant, obtaining a birth certificate from the *Mukhtar,* registration at the *Nofus* (the Personal Status Department), and registration at the Foreigner’s Register. The last step requires valid residency documentation, and many Syrian refugees in Lebanon do not have valid residency [32]. Additionally, many parents do not have a valid proof of marriage, which is required to complete some of the initial birth registration steps such as obtaining a birth certificate from the *Mukhtar* [32].

A number of local and international NGOs and UN agencies are trying to address child marriage in Bekaa area under their child protection, GBV and legal aid programs. However, these programs are reaching a small segment of the population, which is much lower than their required target population [33].

### Data collection

The data for this study was collected through Focus Group Discussions (FGDs), and key informant interviews (KIIs), by three researchers (two females and one male) familiar with these Syrian communities.

The first author (RM), a Syrian post-doctoral research fellow, who is familiar with the research context and who has good experience with qualitative research methods conducted all FGDs with female participants and all KIIs. The female research assistant (OS), a Lebanese who lives in Al Marj and who is familiar with the research context, facilitated the logistics of conducting the FGDs. A male research assistant who worked with an NGO in the area, who is familiar with the context, and who has previous experience in qualitative research, conducted the FGDs with the male participants. The male research assistant was trained by (RM) on ethical research conduct and the study questions one week before data collection started and followed up closely with (RM) while collecting data.

### Sampling

Participants in FGDs and KIIs were recruited purposively by the first author (RM) with the help of the municipality, a local nurse and the two local research assistants. We used purposive sampling, which aimed to include married and unmarried Syrian refugee females of two age groups as well as mothers and fathers of married and unmarried refugee females belonging to those age groups.

Female participants living in the camps/settlements were approached face to face by (RM) and (OS). The municipality simplified the researchers’ entry into three large camps/settlements with the help of the local leader “*the Shawish*”. The municipality also introduced the researchers to a local nurse who is familiar with the Syrian refugee community and who helped with recruiting participants. The researchers, (RM) and (OS), identified eligible participants with the help of the local nurse, explained the study and elicited consent from those interested to participate. Recruitment of fathers was done by the male research assistant and the process of recruitment was similar to that of recruiting female participants living inside the settlements.

Female participants living outside the settlements were recruited purposively by the local research assistant (OS) and the local nurse who knew the Syrian community living in that area and who identified potential participants and contacted them using telephone and text messaging. The researcher (RM) met face to face with the potential participants, identified eligible ones and explained the study.

A verbal consent for participation and permission to make an audio recording of the discussion was solicited from those who agreed to participate in the FGDs as many women were illiterate. The process of obtaining the verbal consent was witnessed by the local nurse and the female research assistant.

To ensure that our sample included a variety of service providers, they were identified by the local research assistant (OS) with the help of the municipality and the local nurse. Service providers were then contacted by (RM) using telephone as a first step followed by a face to face meeting. A written consent for participation and permission to make an audio recording of the discussion were obtained from all service providers who agreed to participate.

### Focus group discussions

Eight FGDs, each comprising 6–8 people of the same gender, were implemented with young women and parents. Only young women 18 years of age or older were included in these discussions, in order to ensure proper consent procedures. At the time of the study in 2014, younger married girls (18–19 years of age) who married before turning 18 were assumed to have married after the conflict, which began in 2011; older married girls (22–24 years of age) who married before turning 18 were assumed to have married before the conflict. Groups included the following (Table 1).

**Table 1:** A brief description of the FGDs conducted for this study

Group	Participants	Recruited
18–19 year old women; married	6	from inside the settlements
18–19 year old women; unmarried	6	from inside the settlements
22–24 year old women; married	6	from inside the settlements
22–24 year old women; unmarried	6	from outside the settlements
Mothers of 18–19 year old daughters; unmarried	6	from outside the settlements
Mothers of 18–19 year old daughters; married	6	from outside the settlements
Fathers of 18–19 year old daughters; unmarried	6	from inside the settlements
Fathers of 18–19 year old daughters; married	8	from inside the settlements
TOTAL	50	

There are a number of reasons why we chose FGDs to collect data. FGDs are often recommended as a standalone method, for research relating to group norms, meanings and processes [34]. The group interaction can help in generating a rich understanding of participants’ experiences and beliefs [34]. When comparing FGDs with individual interviews: “Group discussions provide direct evidence about similarities and differences in the participants' opinions and experiences as opposed to reaching such conclusions from post hoc analyses of separate statements from each interviewee” [35]. There are opposing views in the literature regarding the suitability of FGDs to discuss sensitive topics, but one of the downsides of FGDs is that the presence of other participants may compromise confidentiality and discourage some participants from discussing private or sensitive topics. Despite this limitation, we chose FGDs to collect data because they provide a good platform for addressing social norms and changes affecting entire communities, such as the impact of the conflict and forced migration.

After giving their consent, participants in FGDs were asked about a number of topics related to their current livelihoods and marriage practices before and after the conflict. The duration of the FGDs ranged between 45 min and 60 min.

Adolescent girls were asked about their current situation and vision of the future, their current life, their hopes and expectations, and if these changed after the conflict. They were also asked about marriage practices, their perceptions of them and how practices and perception of marriage had changed since conflict and displacement. Adolescent girls were also asked about services that they need most.

Parents of adolescent girls were asked about challenges that they and their daughters face during displacement, marriage practices, factors that encourage girls to marry before turning 18, and the positive and negative aspects of child marriage.

FGDs with participants living inside the settlements took place at a common meeting place, one big tent, within the settlement. FGDs with participants living outside the settlements took place at the Mayor’s place and at a local health centre. Participants had easy access to both locations. All women we approached agreed to participate and there were no dropouts. It was difficult to recruit fathers of 18–19 year old women as many were reluctant with the use of a tape-recorder so these interviews were hand written and there were no dropouts.

### Key informant interviews (KIIs)

Eleven key informant interviews were conducted including:

- Four local and international NGOs in the field of child protection
- Two government officials in Al Marj community
- One camp leader (*Shawish*)
- Two religious officials: a Syrian male religious leader at one of the camps, and a Syrian female religious mentor for Syrian refugee women
- One Syrian female schoolteacher who teaches Syrian refugees at a private school
- One female gynecologist who treats Syrian refugees

After giving their consent, respondents were asked a set of piloted semi-structured questions. One interview was conducted in English and the rest of the interviews were conducted in Arabic. The duration of interviews ranged between 45 min and 60 min.

Respondents were asked about the most common issues facing adolescent Syrian women in Lebanon, customs and norms relating to marriage among Syrian refugees, and the main causes and consequences of child marriage.

The in-depth interviews were conducted either at the workplace of the participants or at their home. All service providers we approached agreed to participate and none of them dropped out.

### Data analysis

All interviews and FGDs were transcribed verbatim by a research assistant. Thematic analysis was used for data analysis, following Clarke and Braun’s six phases of thematic analysis; familiarization, coding, searching for themes, reviewing themes, defining and naming themes and writing up [36].

One of the investigators listened to the interviews a number of times noting initial impressions. In the second step, a general codebook was developed according to the main domains of inquiry and the transcribed data was rearranged and summarized according to the descriptive codes. In the third step, sub-themes derived from the findings were identified where recurring themes across the three groups of interviewees were arranged under the corresponding main themes. Finally, relevant quotes were translated into English.

The authors were not able to validate the findings with the respondents, as no direct identifiers of participants were obtained. Additionally, this is a highly mobile population that does not always have a fixed address or clear contact details, so it would not be easy to follow-up with respondents. However, the authors obtained feedback on the results from a Syrian public health professor as well as a local health provider who is familiar with the context.

## Results

Most FGDs participants recruited from outside the settlements came from Damascus and rural Damascus while most FGDs participants recruited from inside the settlements came from Deir Ezzor governorate. The majority of participants had been in Lebanon for at least one year, and some had been in Lebanon for three years. Table 2 presents the main themes and subthemes of this study.

**Table 2:** The main themes and sub-themes

Main themes	Sub-themes
A- Challenges faced by refugees	1- Financial pressures and inability of families to meet basic needs 2- Loss of educational opportunities 3- Fears are heightened around protection and honor for adolescent girls
B- Change in marriage practices	1- Changes in how girls learn about marriage and relationships 2- Perceptions about age at marriage 3- Challenges to marriage registration 4- Lower bride price 5- Shorter engagement period 6- Change in cousin marriage practices
C- Knowledge about negative consequences of child marriage	1- Inability to register births 2- Difficult living conditions 3- Negative health and social consequences
D- Current services are not meeting women’s needs	1- Lack of trust in Lebanese service providers 2- Services are not always culturally appropriate 3- Inadequate implementation of local programs

### Challenges faced by Syrian refugees

Respondents in FGDs discussed a number of negative consequences of conflict and displacement that imposed additional challenges to the experiences that adolescents normally face. All respondents emphasized that the main stressors they experienced as refugees were inability to meet basic needs, interruption of education for adolescents and insecurity.

### Financial pressures and inability of families to meet basic needs

Conflict and displacement were significant challenges to refugee families. All participants in FGDs reported dire living conditions, poverty, and insecurity. Women emphasized that war and displacement have worsened economic conditions, making daily survival extremely difficult.

The majority of FGDs participants complained that their living situation was unhygienic and cramped, with small tents or rooms shared by many family members. Household finances were described to be dire and likely to become worse. Nearly all respondents expressed their desire to work to support their families. Most respondents described financial pressures due to high rental rates, low wages, and lack of work opportunities. The majority of jobs were unstable or inconsistent. One participant explained:

*“We work one day and stop the next day. Life is expensive and the Lebanese landowners are exploiting us by demanding higher rents. Where are we going to get the money from? This has caused us major psychological distress.”* (*FGD with married 22–24 years old women)*

Financial strain pushed some families to marriage decisions at earlier ages, according to parents and service providers. One woman who worked at a local NGO elaborated:

*“Some people marry off their daughters at an early age because these are their traditions but others do it to ease the financial pressure. For example, there are fathers who are jobless and who have to provide for 5 or 6 people, so marrying off one of their daughters will help.” (KII, NGO)*

### Loss of educational opportunities

Interruption of education was reported by all mothers and service providers as one of the major challenges faced by adolescent girls resulting from the conflict. Young women, especially those who were unmarried, were particularly distressed, expressing that the war had disrupted their education and compromised their future. Many had prior hopes of a college degree and a career, however, now they focused on finding any job to support their families, especially when male family members remained in Syria, died or had been missing due to conflict.

The inaccessibility of education may have encouraged many women to marry before turning 18, and may have pushed many parents to marry off their daughters at an earlier age.

*“We were all pro-education; the priority was education before marriage. We wanted our daughters to reach at least the secondary education level. Things have changed now. Many young girls are resorting to early marriage due to their fear of the ambiguous future. I married off two daughters after the war, one was 18 and the other one was 12.” (FGD with mothers of 18–19 year old married women)*

*“We wished to study and get married, but now the situation is different.” (FGD with 18–19 years’ old unmarried women)*

Married women appeared less concerned about education, possibly because they stopped school once they married or became pregnant, and they were expected to take care of their husbands and children.

### Fears are heightened around protection and honor for adolescent girls

Although many FGD participants expressed that they were against child marriage in principle, some listed advantages of such a practice, particularly in the time of conflict and displacement. The concept of “*al Sutra,*” which means “the protection of the woman’s honor or reputation” in this context, recurred consistently throughout the FGDs as well as the KIIs. Adolescent women’s reputation, as well as that of their families depend greatly on maintaining the women’s virginity until they marry. According to parents and some service providers, many adolescent women were subjected to unfavorable gossip from the local community due to rumors about the presumed social misconduct of some Syrian women. Moreover, they were also vulnerable to sexual harassment, and occasionally even rape due to living in unfamiliar and sometimes insecure areas and the breakdown of social networks. According to most parents, “*Al Sutra*” was the most pronounced factor why parents marry off their daughters during conflict and displacement. As one of the mothers explained:

*“Fear of insecurity is a major factor. They are marrying early because of al Sutra. We have war. Many women are afraid of being raped, and if a married woman is raped, she is more likely to be forgiven by her husband but if an unmarried woman is raped, it will destroy her life.” (FGD with mothers of 18–19 year old married women)*

One woman who works at an international NGO explained:

“*There are parents who think that early marriage provides al Sutra for their daughters, especially that they perceive Lebanese women as open-minded and they worry about the negative influence Lebanese women might have on their daughters, so they resort to marrying them off at an early age. We have encountered this issue many times.”*

Exposure to Lebanese social norms, which are felt to be more liberal than the Syrian ones, may have encouraged some parents to marry their daughters early. Mothers living outside the tented settlements expressed great concern about their daughters being influenced by the culture and the marriage practices of the Lebanese community. A number of mothers were worried about their daughters’ friendships or marriages with Lebanese men, as they highly doubted these men’s intentions and believed that these marriages are likely to end in divorce.

One service provider, a Syrian female religious mentor, expressed her concern about the consequences of living in Lebanon:

*“There is a change in the values of young women. They are imitating Lebanese women with the way they dress. They are interacting with men. This did not exist in Syria.”*

Refugees living inside the settlements, where women’s restricted mobility results in minimal social exposure, were less concerned about this issue than parents of women living outside the settlements where women often came from urban areas and were used to going out alone.

### Change in marriage practices

Generally, participants in FGDs and KIIs agreed that a number of marriage practices changed as a result of the conflict.

#### Changes in how girls learn about marriage and relationships

There was an agreement among FGDs participants, particularly among adolescent girls and mothers, that adolescent girls learn about marriage from their mothers around the start of their teenage years, and to a lesser extent from female relatives, school and youth centers. However, women outside the tented settlements, those who mostly came from urban areas and who were generally more educated, mentioned that their primary sources for such information are their own experiences and the internet. This was not the case for women living inside the settlements, who had restricted mobility and often did not have access to mobile phones or the internet. Similarly, some mothers who lived outside the tented settlements pointed out that these practices have changed since many adolescents have mobile phones and more global exposure:

*“These days, women learn everything from the internet. It increased our awareness. We never used WhatsApp, Facebook or internet when we were in Syria. We did not even have mobile phones. I was not allowed to have a mobile phone until I started working. We used to be more conservative, though the situation is different now, and each member of the family has a mobile phone.*” (FGD with unmarried 22–24 years old women)

*“The mobile phone is teaching our children everything. They did not have a phone in Syria. We only had a landline and they never used it. This technology had a bad influence on them. You see children as young as 10 using WhatsApp and Facebook.” (FGD with mothers of unmarried 18–19 years old women)*

#### Perceptions about age at marriage

Although many married women got married before turning 20 in Syria, there was a general agreement among young women (married and unmarried) that the minimum age of marriage should be 20 for women and 25 for men despite being displaced. Nonetheless, parents of current adolescents seem to disagree, as most mothers thought that the age at marriage should be lower in a context of displacement, and some stated that they would marry their daughters at the age of 16–17 if the potential groom were suitable and financially independent. Some fathers believed that women should marry at a younger age, as young as 15, and there should be a wider age difference between the bride and the groom than what mothers believed.

*“She should not marry if she is below 18 because she does not understand anything.” (FGD with 18–19 year old women)*

*“The best age for girls to marry is 18-20 year old but if the man is suitable she can get married at 16 or 17.” (FGD with mother of unmarried 18–19 year old women)*

*“Girls should get married at age 15 and above, whereas boys should get married when they are at least 18 and the age difference between the bride and the groom should be 5 to 15 years.” (FGD with fathers of 18–19 year old women)*

#### Challenges to marriage registration

All participants in FGDs agreed that “*Katb Kitab*,” “a registration of marriage under Shari‘a law,” was the only form of marriage acknowledged and accepted by most Syrians in Lebanon. However, many Syrian refugees do not finalize the legal registration of their marriage. A women working at an international NGO explained:

*“Syrians are not banned from registering their marriages in Lebanon. The thing is that people lack information about services, and they do not know where to seek assistance from. There are also many misconceptions; they think that if they register their marriage in Lebanon, they will not be able to register it in Syria. The fees of registration are also another barrier, even if they do not seem too expensive to me and you.”*

One woman complained about not being able to obtain an official birth certificate for her daughter because her marriage was not registered in Lebanon. Although women whose marriages are not registered are able to obtain a hospital birth certificate for their baby, in her case she could not since she delivered at home:

*“We were not able to register our marriages officially. My daughter does not have a birth certificate [from the hospital] because she was born at home.” (FGDs with 18–19 year old married women)*

#### Shorter engagement period

Several participants in FGDs and KIIs explained that the engagement period is becoming shorter

*“The engagement period varies... but in these conditions it only lasts for a month and they tend to marry immediately because the girl does not expect any presents anymore. In addition, most of the girls are out of school so the grooms do not have to wait for their fiancés to finish school as they used to do back in Syria.” (FGD with 22–24 year old unmarried women)*

*“There are no more conditions, no more traditions, no engagement period, they are getting married quickly.” (A female religious teacher)*

#### Lower bride price

There was disagreement about trends in the amount of bride price *(mahr)* being paid after the conflict. Most mothers, women and some service providers reported that the bride price is lower than it used to be before the conflict due to reduced earnings and assets. Others, particularly married 18–19 year old women indicated that the bride price increased after the war due to poverty. In all cases, it appears that the amount of the bride price became highly dependent on the nationality of the groom and whether he is related to the bride. The former amount of the bride price varied according to region in Syria, usually ranging between $100 and $500 but sometimes as high as $3000. The bride price after the conflict was estimated at $100 or lower if the groom is Syrian and higher if the groom is Lebanese, however, the grooms are usually Syrian. Bride price amounts were generally lower for relatives, who were often exempted entirely.

*“The bride price varies, for a cousin it is 10000-15000 SP but for strangers it is 20000 to 30000 SP” “if the stranger cannot afford the bride price, he has to work and save money.” (FGD with married 18–19 year old women)*

*“Parents are marrying their daughters off or money. I have witnessed many marriages where the girl is 14 or 15 and she gets married for 10000 or 150000 SP.” (One service provider)*

#### Change in cousin marriage practices

Cousin marriages, in some traditional societies, involve family loyalty and knowledge of the background and the upbringing of the cousin, and are thus viewed as more protective. Participants in FGDs had different opinions regarding cousin marriage practices after displacement; although a few participants explained that the practice was still consistent, the majority asserted that the practice appears to be declining during displacement due to heightened awareness about its negative consequences, the increased tension between families resulting from living in cramped households, and the dispersion of many families, which was caused by war and displacement.

*“We no longer have cousin marriages. People are more aware these days about the negative pregnancy outcomes resulting from cousin marriages.” (FGD with mothers of unmarried 18–19 year old women)*

*“Cousin marriages are not desired because people are living with their extended families and this is causing conflict, plus most families became scattered.” (FGD with 22–24 unmarried women)*

### Knowledge of the negative consequences of child marriage were high

Despite their confidence that there are positive advantages of child marriage in the context of displacement, some parents, particularly those who did not marry off their daughters, demonstrated high awareness of the negative consequences of child marriage.

Interestingly, married women were more likely to discuss those negative consequences than unmarried women. In addition to their inability to register their births as a result of unregistered marriages, married women also discussed their difficult living conditions.

*“It is wrong for women to marry in these times. We all lost our houses in Syria, where will they live? In the camps?” (FGD with married 18–19 year old women)*

One of the most important negative consequences of child marriage stated by service providers, and some parents, was the negative health consequences of early marriage on mothers and their babies, which included early pregnancy and negative psychological consequences.

*“I married off my daughters before they turned 18 but felt sorry for them because they had to assume physical and emotional responsibilities at an early age.” (FGD with mothers of 18–19 year old women)*

The question posed by several parents in FGDs was:

*“How can she take care of her baby? She is a child with a child.”*

### Current services do not meet women’s needs

#### Lack of trust in Lebanese service providers

Women, particularly those who were married, expressed their needs for certain services like vocational training and education, easier access to health services, medications and vaccinations for their children. However, women also expressed their lack of trust in Lebanese service providers due to being treated badly on many occasions. A woman explained:

“*How can they offer us emotional support if they cannot even stand us? I doubt that they will accept to receive us.” (FGD with married 18–19 years old women)*

A few service providers emphasized the need to gain the Syrian refugees’ trust before any attempt to raise their awareness about child marriage. They elaborated that this can be achieved by proceeding slowly, and attempting to get to know the refugees and gain their trust before starting with the awareness sessions. One woman noted:

*“You cannot simply tell them this is wrong, you need to approach them slowly and you need to be patient as it might take long to reach the desired result but thank God we are achieving results in the areas where we are working.”*

#### Services are not always culturally appropriate

Many awareness sessions are provided to mothers and girls. However, since the main decision maker is typically the male head of the household, many of the awareness sessions do not achieve desirable results as reported by some service providers. For instance, one woman who worked at a local NGO illustrated:

*“I had a woman who attended our awareness sessions for 8 months, yet she wants to marry off her young daughter. It is their mentality, and the decision is not theirs. In Syrian families, usually the man decides, especially for people who come from rural areas. There should be awareness sessions for men, but I doubt they will be effective. These are deep-rooted traditions inherited throughout generations.”*

Another woman who worked at an international NGO explained:

*“From my limited experience, I believe that awareness sessions on child marriage are not making a difference. It is hard to change this practice. We are talking here about deep-rooted traditions. To prevent child marriage effectively, you have to reduce the burdens of refugees. Enrollment in schools, vocational training for girls and empowerment are some examples that can decrease child marriage.”*

#### Inadequate implementation of local programs

A number of service providers highlighted the fact that it is hard to reach Syrian refugees, as many refugees do not attend awareness sessions provided at NGO centers for a number of reasons including lack of knowledge about those services, inability for women to leave the settlements and inability to pay for transportation. Therefore, some service providers suggested a number of strategies to increase access to Syrian refugees such as sending mobile teams from the centers to reach women who cannot leave the settlements, providing awareness sessions in safe places (common meeting places such as praying rooms) within the settlements, or offering transportation for women willing to attend the sessions.

*“You can either conduct awareness sessions at the settlements or invite adolescents' of the ages that you want to target to attend awareness sessions at NGOs centres. As for women who married early, you can reach them through giving them financial incentives to attend awareness sessions. They only attend when there are incentives.” (KIIs, NGO)*

## Discussion

This study reveals that there appear to have been changes in marriage practices and in the risk of child marriage as reported by young women, parents of women and service providers in Lebanon. While our study cannot report on trends, it provides qualitative data suggesting that drivers of child marriage appear to have been exacerbated by the conflict and forced displacement. This research confirms many reported drivers of child marriage among Syrian refugees, such as concerns over safety, worsening economic conditions, and disrupted education for adolescent girls that have been reported elsewhere but are different in a context of displacement [13–16].

One of the major themes that emerged was the importance of *“al Sutra,”* the social protection and preservation of a girl’s honor. This was cited as a main driver of child marriage by nearly all interviewees, including women, parents and service providers. In traditional Arab societies, a girl who loses her virginity brings dishonor to her family [37, 38]. According to this research, war and displacement increased refugees’ sense of insecurity and vulnerability and their real and perceived risks of sexual harassment. Thus, they felt an increased need to protect their daughters and their family’s honor, and many parents opted for child marriage as a means of doing so.

One of the findings that distinguishes this research from prior work, and which was unexpected, is the observed differences between refugees living in tented settlements and those living outside the tented settlements. Within tented settlements, refugee communities who were mostly from rural areas reported less heightened concerns that would lead them toward child marriage. There are several factors that may underlie this result. These communities were more insulated. Inhabitants came from the same region, were able to preserve community ties and therefore perceived fewer threats to women. Women’s mobility was severely restricted, thus minimizing contact with Lebanese society. Furthermore, some of the communities of origin where these women came from supported cousin marriages, so marrying for financial reasons was not as prevalent among those communities. Finally, many of the women inside the tented settlements had discontinued their education before coming to Lebanon, so the disruption of education was less significant as a risk factor for child marriage. Thus while living standards were poor within tented settlements, communities were more able to maintain their traditions and value systems.

Refugees living outside the tented settlements, however, had greater concerns about their daughters’ prospects in ways that would push them toward child marriage. These refugees came from more urban, wealthier communities in Syria, where most young girls and many of these families expected the girls to proceed for a full education and even careers. Most of these young women and their parents were aware of the negative consequences of child marriage. They might have perceived that the risks of having an unmarried woman or being unmarried in these circumstances exceeded the risks of marrying early.

Women outside settlements experienced greater physical, financial and social threats than women living inside the settlements, especially with the limitation of education and work opportunities and the increased exposure to and the influence of the Lebanese society, thus potentially leading them to seek security in child marriages. Furthermore, girls themselves may have resorted to child marriage to face the future with a greater security and a new purpose in life.

This study also highlights the constraints women experience in registering their marriages legally in Lebanon, particularly women living inside the tented settlements. When marriages are not registered, this has implications for birth registration and for access to services for the newborns, and eventually for school enrolment and ultimately for the life chances of children of these marriages.

Although our study is small-scale and not representative of the Syrian refugee community in Lebanon, it is one of the first qualitative studies among Syrian refugees in Lebanon and highlights some important issues. Drivers of child marriage in this conflict and displacement setting appear to be very different from or more intensified than those in stable settings because of the changing nature of marriage, economic hardships, and heightened concerns about daughters’ safety in the new environment. Additionally, challenges faced after displacement seem to differ according to where refugees came from in Syria and where they settled after displacement. These differences have significant implications for program interventions in these different communities.

There are many studies in the literature that discuss programs aimed at decreasing child marriage, but the majority are in non- conflict settings. A recent systematic review of 83 papers that evaluated interventions addressing issues related to child protection, including child marriage in low- income settings, revealed that successful interventions often used a combination of approaches to decrease child marriage practices [39]. Such approaches encompassed empowering girls with information, skills, and support networks, mobilizing communities, enhancing access to education, offering economic support and influencing legal policies. Addressing security was not suggested, as these studies took place in non-conflict settings.

A successful intervention would require: 1- identifying drivers of child marriage in conflict and displacement settings and 2-addressing those drivers concurrently in ways that work in such settings. This in turn, would require the engagement of, and collaboration between multiple stakeholders.

Our findings highlighted three main drivers of child marriage in this setting, which were also highlighted in other humanitarian settings: 1- interruption of education, 2- economic hardships and 3-lack of security. A recent review of child marriage in the Arab region as well as in humanitarian settings suggested some future strategies that address those drivers, and these include increasing girls’ access to education, establishing awareness raising campaigns on the negative effects of child marriage, offering health and education sessions to married girls and other measures at the policy level such as proper law enforcement [13]. The review also summarizes the strategies suggested by three reports: one that focuses on child marriage globally [40], one that focuses on child marriage in the Arab region [41], and one that discusses child marriage in Yemen [42]. Those reports suggested working with girls and communities in order to increase girls’ access to education, provide them and their families with economic support, mobilizing communities to mitigate the impact of child marriage, improving sexual and reproductive health services, including empowerment programs in humanitarian settings, and finally fostering enabling legal environment [13].

Many NGOs adopted intervention programs that appear to focus on raising awareness trying to address traditions as the main driver of child marriage, without taking into account other drivers of child marriage, or its negative consequences, particularly in the context of conflict and displacement. Interventions that aimed at increasing school enrollment among refugees failed to prevent school dropouts [43] or to address the needs of the most vulnerable adolescents aged 15–18 years old [44]. Moreover, there is a strong need to mitigate the negative effects of child marriage and encourage legal registration of marriages – which has knock on effects for the next generation. Current established child marriage programs fail to take into consideration that traditional drivers of child marriage may be influenced by additional factors that are related to conflict, such as displacement, mobility and insecurity.

Recommendations to decrease child marriage practices should take into consideration women’s needs, cultural background, and their status as refugees. Programs aimed at decreasing child marriage should be designed according to the issues faced by the different groups of Syrian refugees. For those living inside the tented settlements, the focus should be on interventions that address traditions that contribute to child marriage practices, and raise awareness about its negative consequences. Such awareness sessions should particularly target men, as male family members tend to be the main decision makers in such communities. Additionally, such interventions should take into account the cultural background of those communities, their financial pressure, as well as the lack of security, which may prevent many women from attending the awareness sessions offered at NGOs centers. Outreach awareness sessions could be useful in these situations. For Syrian refugees outside the tented settlements, interventions should initially focus on meeting their basic needs by offering economic support through vocational training with skills that match the job market, increasing their access to education and supporting them to stay in school, and enhancing security by collaborating with local municipalities.

Although UNHCR is adopting a multi-sectorial approach in addressing child marriage in some settings such as Algeria [45], such an approach, to our knowledge, is not yet being explicitly addressed in Lebanon, possibly due to the additional challenges that Lebanon is facing with its limited resources, and its political and security challenges. Moreover, humanitarian agencies are more likely to prioritize meeting basic needs such as food, shelter and water, especially in a setting with limited resources. Additionally, the potential interlinkages between child marriage and education, employment, housing and other issues are not always being made in the response to the Syrian refugee crisis.

Interventions should not be limited to addressing drivers of child marriage but also they should attempt to mitigate its negative effects for women who are already married, yet very few NGOs are addressing such issues. For instance, the Norwegian Refugee Council has been offering legal advice to Syrian refugees on how to register marriages in Lebanon officially, as well as births [46]. However, there appears to be little coordination between NGOs working on such issues.

### Limitations

This study is qualitative; therefore, the objective was not to generate findings that are representative of all the Syrian refugees living in Lebanon but rather to learn insights about perceptions about child marriage. All interviewed women were above 18, and participants in this research came from two governorates in Syria: Deir Ezzor and Damascus. It would have been more informative to recruit participants from other areas in Syria, and to recruit women who were below 18. However, this was not possible due to time constraints.

Another difficulty was finding the minimum required number of women and parents within one tented settlement of the required age and marital status. Although none of the women we approached refused to participate, many of those women refused to leave the settlements where they lived to join another FGD in another settlement, which made it difficult for us to secure the minimum number of participants in a FGD. As a result, participants were additionally recruited from outside the settlements highlighting an important but unforeseen issue: the differences in the challenges faced by women living inside and outside the tented settlements.

Although we anticipated that the recruited 18–19 years old married women got married after the conflict, and the 22–24 years old married women got married before the conflict, not all women matched these assumptions. One woman in the first age group got married at 18, and several women in the second age group were married after the conflict.

It was extremely difficult to recruit fathers of 18–19 year old women, as many men were reluctant to be formally interviewed. To encourage participation, these interviews were not recorded, and were thus less detailed than those with women.

### Conclusion and recommendations

The main aim of this study was to explore drivers of child marriage in the context of conflict and displacement and to generate recommendations on how best to address child marriage. This study highlighted three important issues: change in marriage traditions and practices after the conflict, the additional factors that could contribute to child marriage practices during conflict and displacement and the differences in challenges faced by refugees living inside and outside the settlements.

Although other studies may have demonstrated similar issues, each national situation of child marriage is unique, and addressing such issues would require a tailored approach. To be able to mitigate drivers and negative consequences of child marriage effectively, there is a need to understand drivers of child marriage in conflict settings and address them through working with different members in the community including parents, teachers, health workers and religious leaders, both to prevent child marriage and to support those who are already married. Interventions should be multi-sectorial and adapted to each specific context, age group and marital status, and the mode of delivering such interventions should be practical and culturally acceptable, using contextually appropriate strategies to ensure access to hard- to -reach refugees. These can only be achieved through extensive interactions with the community to understand their backgrounds, their needs and to gain their trust. Such a multi-sectorial intervention is currently being planned as a follow up of this study.
